# Environmental Investigation of Natural Radioactivity and Health Risk Assessment in Basaltic Volcanic Building Materials

**DOI:** 10.3390/toxics14010015

**Published:** 2025-12-22

**Authors:** Turki Kh. Faraj, Ahmed E. Abdel Gawad, Mayeen Uddin Khandaker, Mohamed Y. Hanfi

**Affiliations:** 1 Department of Soil Science, College of Food and Agricultural Sciences, King Saud University, Riyadh 11451, Saudi Arabia; 2 Nuclear Materials Authority, El-Maadi, P.O. Box 530, Cairo 11381, Egypt; 3 Applied Physics and Radiation Technologies Group, CCDCU, Faculty of Engineering and Technology, Sunway University, Bandar Sunway 47500, Selangor, Malaysia; 4 Faculty of Graduate Studies, Daffodil International University, Daffodil Smart City, Birulia, Savar, Dhaka 1216, Bangladesh; 5 Department of Physics, College of Science, Korea University, 145 Anam-ro, Seongbuk-gu, Seoul 02841, Republic of Korea; 6 Department of Life Safety, Institute of Fundamental Education, Ural Federal University, St. Mira, 19, 620002 Yekaterinburg, Russia; 7 Department of Physics, Dogus University, Dudullu-Ümraniye, 34775 Istanbul, Türkiye

**Keywords:** basaltic volcanic rocks, uranium-thorium-potassium, environmental risk, statistical analysis, building materials

## Abstract

This study presents an integrated geological and environmental radiological analysis of basaltic volcanic rocks, which have been characterized by their suitability and potential for risk when used as construction materials. A total of thirty-five representative basaltic samples from the environment of studied area, located in the Northern Eastern Desert of Egypt, were utilized for this study. The rocks were then analyzed by means of HPGe high-resolution gamma-ray spectrometry methods. The petrographic studies show that the basalt samples were composed mostly of three main minerals: plagioclase, olivine, and pyroxene. In addition, these rocks have a significant degree of secondary alteration products, including sericite, epidote, and zoethite. For uranium-238 (^238^U), thorium-232 (^232^Th), and potassium-40 (^40^K), the average activity concentration measured 53 ± 20 Bq kg^−1^, 54 ± 14 Bq kg^−1^, and 1178 ± 269 Bq kg^−1^, respectively. Using the current global reference limits, all the measured values are above acceptable levels for the radionuclides ^238^U, ^232^Th, and ^40^K. The radiological indices calculated for each of the basalt volcanic samples measured radium equivalent activity (Ra_eq_ = 221 Bq kg^−1^), external hazard index (H_ex_ = 0.60), internal hazard index (H_in_ = 0.74), gamma index (Iγ = 0.84), and annual effective dose (AED = 0.52 mSv y^−1^) indicate that the radiological hazard values of these samples are acceptable, unlike several samples, where values are near or exceed the accepted standards for indoor hazards. The most significant finding of this study reveals that the major contributions in the environment from radiological risk can be attributed to radionuclides ^238^U and ^40^K based on correlation analysis, hierarchical clustering, and PCA analyses, and this study establishes the first multivariate perspective of how radiogenic materials controlled by the environment can affect basaltic rocks. Therefore, this study creates an important baseline for future environmental monitoring and states that caution is warranted when using basalt as a finished material for constructed environments, and for using basaltic products as raw materials in indoor environments.

## 1. Introduction

Natural radioactive materials are part of the Earth’s crust, originating from the decay of primordial radionuclides of uranium-238 (^238^U), thorium-232 (^232^Th), and potassium-40 (^40^K). The distribution of these elements varies from one rock type to another and also with regard to the geological setting in which they were created; therefore, the concentration will vary depending upon the geochemical behavior and mineralogical associations, and the tectonic environment. They are present at natural background concentrations, but high levels of these radionuclides can present environmental and health risks, particularly if these rocks are incorporated into human construction or activities [1,2,3]. Basalt volcanic rocks contain macroscopic crystals of plagioclase, olivine, and pyroxene, which have an effect on mineralogical composition and naturally occurring radionuclide distribution. Basaltic rocks are generally considered to be mafic (high in iron and magnesium) and, as a result of partial melting from the mantle, form as low viscosity extrusive. Although they are commonly considered as being “low” in radioactivity relative to granitic rocks, basalts can still contain detectable abundance of natural radionuclides resulting from the presence of accessory minerals and variable geochemical environments during formation [4,5,6]. Basaltic rocks are crucial volcanic rocks, having several applications in many industries. Their properties, including durability, hardness, resistance, and ability to hold a polish, make them valuable for many purposes. They are well used as aggregate for constructing materials of roads, highways, road gravel, concrete mixtures for constructing railroads, bridges, building facades, flooring, pavements, walls, bricks, dust fertilizer, filter stones in drainage projects, pipes, and rebars that are utilized in wind turbine blades. These rocks are used as gemstones in jewelry such as beads or cabochons, statues, monuments, sculptures, ancient artifacts such as Ica Stones Hoax, countertops, tiles, other decorative surfaces for bathrooms and kitchens, garden pathways, rock gardens, headstones, cutters, cemetery markers, memorial plaques, water features, gravestones, grave markers, fountains, fireplace surrounds, aquariums, and terrariums [7,8,9].

The Earth’s crust, the very foundation upon which human civilization is built, is composed of different rocks. The Egyptian rocks are distinguished by their durable ornamental and building stones worldwide, particularly marked varieties of granites, trachyte, rhyolite, imperial porphyry, marble, limestone, sandstone, and gypsum. These rocks are one of the most famous ornamental stones characterized by their sparkling appearance, and used in many applications, such as cladding, ceremonial bathtubs, statues, sculptures, fine objects of art, funeral monuments, baths, paving, vessels, pedestals, bowls, mantle pieces, benches, sarcophagi, and flooring [10,11].

The composition of the source magma, the degree of partial melting, the degree of fractional crystallization, and post-eruption alteration processes are all factors controlling the geochemistry of basaltic rocks. These parameters may lead to enrichment or depletion in uranium, thorium, and potassium in various basalt flows. Finally, basalts erupted in continental or crustal-influenced environments may have high Zr/Nb ratios in contrast to those produced at mid-ocean ridges or hotspots [12,13]. Understanding radionuclide concentration and distribution in basalts is not only crucial to environmental monitoring, but also to geochemical and petrological knowledge. Uranium and thorium, for example, are heat generation and evolution tracing elements in the crust, while potassium is of particular significance for radiometric age determination and estimation of mantle source characteristics. Mobilization of such elements in basalts can hence provide greater insights into processes with magmatic, tectonic settings, and behavior of volcanic provinces on a geological timescale [14,15,16,17].

Basalts are commonly used in construction, road aggregate, and decorative stone in areas with high levels of volcanic activity, highlighting the importance of assessing their natural radioactivity before using them. When these materials contain high levels of natural radioactivity, they can become long-term sources of ionizing radiation exposure, particularly indoors. Their elemental composition should, therefore, be evaluated not only geologically but also from a regulatory and public health perspective. This is especially important in countries with growing infrastructure development in volcanic areas [4,18,19,20,21].

Although various estimates have been made of natural radioactivity in granitic and sedimentary rocks, relatively fewer have been made on basaltic volcanism, particularly where they are widespread in the local geology. As ubiquitous as they are and utilized in as broad a range of applications, there is clearly a need for properly designed investigations that quantify the concentration of radionuclides in basalts and examine their controls on distribution. Such investigations are important in enhancing the improved understanding of the natural radiation background across various fields of geology [22,23,24,25,26,27]. This study investigates the activity concentrations of ^238^U, ^232^Th, and ^40^K in 35 basaltic samples collected from a representative volcanic field. It aims to evaluate the natural variability of radionuclide content between samples and to evaluate their relationships statistically through the application of statistical techniques. The findings of this research will provide a valuable baseline for subsequent geological, environmental, and risk assessment studies in basaltic terrains.

## 2. Geologic Setting

**Figure 1:**
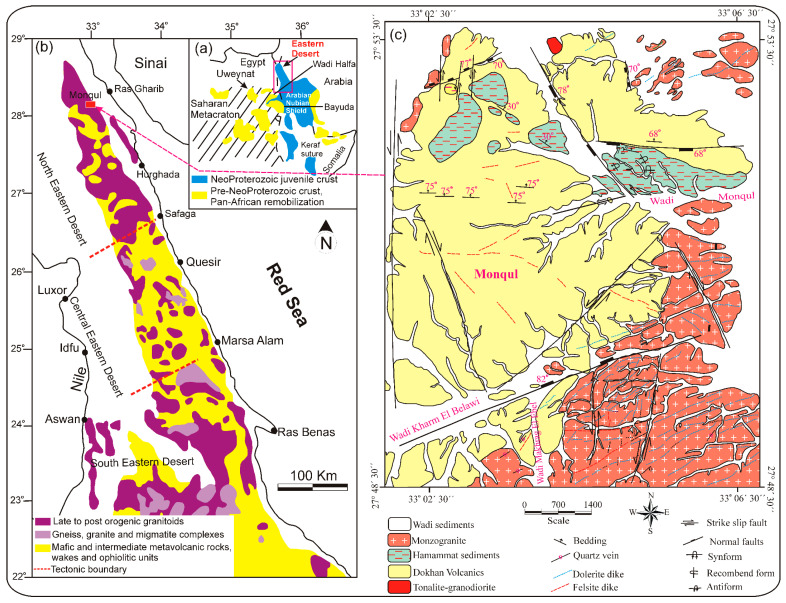
(**a**) Geologic map presenting the ANS; (**b**) geologic map showing the distribution of the basement complex in the ED of Egypt; (**c**) geologic map of Monqul, NED, Egypt.

The Monqul area in Egypt’s Northern Eastern Desert (NED) is part of the Arabian-Nubian Shield (ANS) as shown in Figure 1a–c [28,29]. This environment has considerable interest that could be restricted to the discoveries of many strategically valuable metals encompassing copper, gold, barite, and molybdenum, and radioactive mineralization in south Monqul at Wadi Makharag El-Ebel [4,30,31].

**Figure 2:**
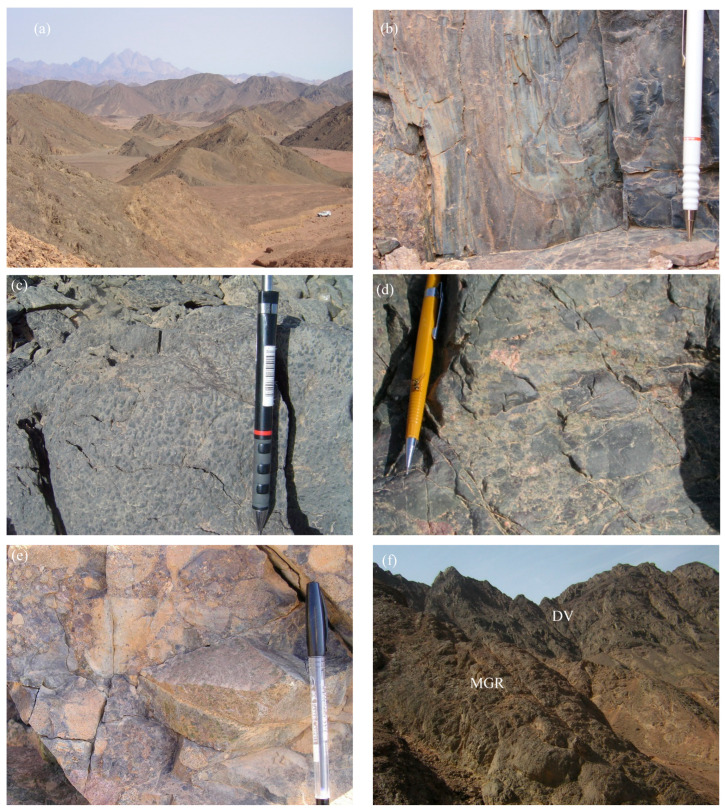
(**a**) Monqul Basaltic Volcanism, (**b**) basaltic lava flow, (**c**) close-up view showing basaltic rocks, (**d**,**e**) close-up views of Hammamat conglomerates vary from basaltic to andesitic composition, (**f**) monzogranite (MGR) intrudes the Dokhan volcanics (DV).

The present work reveals that the exposed basement rocks in the Monqul area comprise tonalite–granodiorite, Dokhan volcanics, Hammamat sediments, and monzogranite, which were invaded by dike swarms including felsite and dolerite dikes, in addition to quartz and barite veins (Figure 2a–f). These dikes having E–W, NE, and N–S trends.

Tonalite–granodiorite occurs as small scattered masses in the northern corner of the investigated area (Figure 1c). They are varied from whitish gray to pale gray colors, and show low relief. These rocks are medium- to coarse-grained, highly fractured, jointed, and altered, and show boulder appearances. They are composed essentially of plagioclase, quartz, K-feldspars, hornblende, and biotite.

Dokhan volcanics are considered the most predominant rocks in the investigated area. They comprise a substantial series of layered lava flows intermingled with their pyroclastics. These volcanics exhibit characteristics of being dense, hard, compacted, and fine-grained, and vary from dark gray, black, green, whitish-gray, buff, reddish-pink, and red colors. Predominantly, they are mainly composed of basaltic (Figure 2a–c), dacite, rhyodacite, and rhyolite, with occasional layers of ignimbrite and deep purple hues of imperial porphyry. The pyroclastics encompass laminated ash-tuffs, lithic tuffs, crystal lapilli tuffs, and agglomerates. The Dokhan volcanics were covered by a substantial sequence of non-conformable manner, the Hammamat sediments along the E–W trend at Wadi Monqul. The Hammamat sediments are composed of basal conglomerate (Figure 2d,e), sandstone, greywacke, siltstones, and silty mudstones.

Monzogranite occurs as medium- to coarse-grained, high relief, and varies from buff to reddish pink and buff colors. It is composed mainly of plagioclase, K-feldspar, quartz, and biotite. This rock is widely distributed in the southern, eastern, and northern parts of the investigated Monqul area, especially at Wadi Makhrag El Ebel. It is strongly altered and enriched in visible Cu-mineralization and barite veins that are predominant in phyllic, potassic, argillic, and prophylitic alterations. Copper minerals that were recorded included chrysocolla, tenorite, chalcopyrite, bornite, enargite, and covellite in addition to gold mineralization [32]. Monzogranite intrudes the Hammamat sediments and Dokhan volcanics (Figure 2f).

## 3. Petrography of Basaltic Volcanism

**Figure 3:**
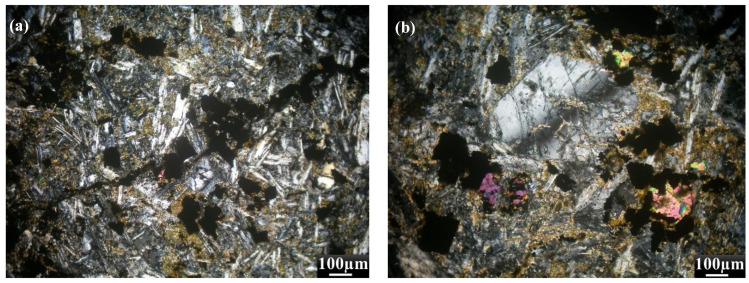

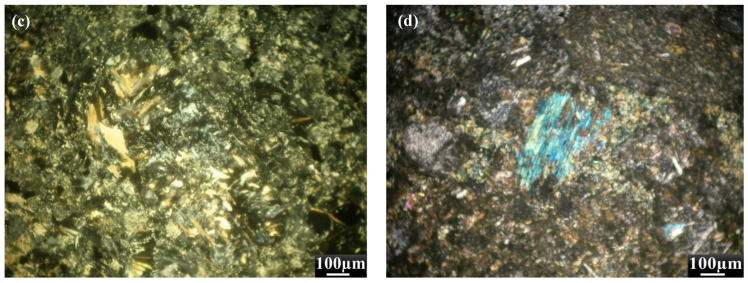
Photomicrographs of the studied Basaltic rocks at Monqul, NED, Egypt, clarifying: (**a**) Sericitized plagioclase with epidote and opaque, (**b**) zoned crystal of plagioclase associated with epidote, (**c**) plagioclase crystal altered to zoethite with opaque, (**d**) pyroxene altered to tremolite. All photomicrographs have been taken under Crossed Nicols (C.N.).

Basaltic rocks are fine-grained, massive, hard, and range between black and dark-gray colors. These rocks are distinguished by amygdaloidal textures. They are holocrystalline and consist mainly of plagioclase, olivine, and pyroxene (Figure 3a–d). Accessories encompass opaque minerals and apatite, while chlorite, tremolite, actinolite, epidote, and zeolites are alteration products (Figure 3a–d). Plagioclase crystals are of labradorite type (An_55–60_), and occur either as phenocrysts or as a major groundmass constituent (Figure 3a). In some samples, amygdales are filled with fine laths of plagioclase, quartz, and opaque minerals. Plagioclase crystals altered to sericite and zoisite (Figure 3c). Olivine occurs as aggregates between plagioclase laths. Pyroxene occurs in subhedral prismatic crystals, fine-grained, pale greenish aggregates, and can be transformed into fibrous tremolite, actinolite, and chlorite (Figure 3d). Opaque minerals occur as anhedral crystals in the fine-grained groundmass (Figure 3a–d).

## 4. Materials and Methods

### Sample Preparation and Measurement

About 35 basaltic volcanic samples had been accumulated from various volcanic outcrops inside the observation area to assess their natural radioactivity levels. The samples were selected with both a wide variety of geological types and minimal human interference. Each sample, with a weight ranging from one to two kilograms, was obtained through the use of non-contaminating tools and stored in clean, categorized polyethylene baggage to prevent cross-contamination. In the laboratory, the basaltic samples have been first washed with distilled water to remove surface dust and impurities, and then dried in an oven at 105 °C for twenty-four hours to remove moisture content. Once dried, the basaltic samples were crushed using a jaw crusher and subsequently ground to a fine powder with the use of an agate mortar or mechanical mill to ensure particle length homogeneity. The powdered samples were then sieved through a 2 mm mesh to acquire a uniform grain length suitable for gamma spectrometric evaluation. Each homogenized sample was located in a 250 mL standard plastic container and sealed tightly to prevent the escape of radon gas. The containers in which the samples were kept were weighed, labeled, and kept in a storage facility for no less than four weeks in order to allow for the establishment of secular equilibrium among radon (^222^Rn) and its decay products in the uranium and thorium decay series. This equilibrium is critical for accurate activity estimation of ^238^U and ^232^Th via their gamma-emitting daughters.

The radiological evaluation of the 35 organized basaltic samples is performed the use of high-resolution gamma-ray spectrometry with a high-purity germanium (HPGe) detector. The HPGe detector is known for its superior energy resolution and efficiency in detecting low levels of gamma radiation, making it perfect for quantifying natural radionuclides in geological materials. The detector used in this study was a coaxial p-kind HPGe detector with a relative efficiency of approximately 40% and a resolution of 1.8 keV full width at half maximum (FWHM) on the 1.33 MeV top of ^60^Co. The spectrometric system changed into coupled with a multichannel analyzer and operated in a low-background lead shielding chamber composed of 10 cm thick lead, internally lined with layers of cadmium and copper to reduce X-ray and Compton background interference. Prior to analysis, the HPGe changed into carefully calibrated for each energy and absolute efficiency via certified reference materials (CRMs) obtained from the International Atomic Energy Agency (IAEA). The calibration utilized IAEA-448 (sediment), IAEA-RGU-1 (high-uranium ore), IAEA-RGTh-1 (high-thorium ore), and IAEA-RGK-1 (potassium sulfate), each with known ^238^U, ^232^Th, and ^40^K activity concentrations. These requirements have been selected to match the geometry, density, and matrix of the basaltic samples, thereby ensuring high accuracy in the efficiency calibration and dimension technique. Each powdered sample was packed into a standard plastic container and counted for a stay time of 86,400 s (24 h) to achieve the highest quality detection sensitivity and statistical reliability. Background radiation was monitored frequently using an empty container under equal geometry and subtracted automatically from the sample spectra the usage of gamma spectroscopy Genie 2000 software program. The specific activity concentrations of ^238^U, ^232^Th, and ^40^K have been determined with the aid of analyzing the characteristic gamma emissions from their decay products. For ^238^U, peaks from 609 and 1120 keV were used; for ^232^Th, emissions from 583 keV and 911 keV were analyzed. ^40^K activity concentration was measured the use of its distinguished gamma line at 1460.8 keV. Calculations were based on the subsequent formula [33]:

(1) $$A(\mathrm{Bq}\;{\mathrm{kg}}^{-1})=\frac{C}{\varepsilon \cdot P\gamma \cdot m}$$

where the specific activity concentration of radionuclide labeled by way of (A), C is the net count rate under the gamma peak (counts per second), ε is the HPGe’s performance in uniform gamma efficiency, Pγ is the probability of gamma radiation emission, and m is the sample’s mass in kg. The Minimum Detectable Activity (MDA) affords the minimal amount of radionuclide that can be reliably measured. To calculate the MDA, the method used was the Currie detection limit formalism developed in 1968. The method is based on background radiation’s Poisson distribution. The constants 2.71 and 4.65$\sqrt{B}$ in this theory provide the detection limits (4.65$\sqrt{B}$) and critical limits (2.71), where B is equal to the background count, describes the background radiation count for the particular energy peak of interest. In all cases, this approach provides a statistically meaningful detection rate at 95% confidence. The MDA is estimated through the following formulation [33]:

(2) $$MDA=\;\frac{2.71+4.65\sqrt{B}}{\varepsilon \cdot P\gamma \cdot t\cdot m}$$

In the context of this evaluation, “B” shows the background count rate, and “t” denotes the duration of the counting procedure, measured in seconds. For this configuration, the standard MDA values have been 2, 4, and 12 Bq kg^−1^ for ^238^U, ^232^Th, and ^40^K, respectively. The values indicated herein characterize the detection sensitivity for every radionuclide, as determined under the precise conditions of the analysis. Furthermore, the dimension uncertainties are expressed in terms of the standard deviation (±2σ). This standard deviation was derived using the following equation: σ = $\sqrt{\frac{N_{S}}{T_{S}}+\frac{N_{b}}{T_{b}}}$. In the context of the present study, N_s_ and T_s_ are defined as the sample counts and measurement time, and N_b_ and T_b_ are the corresponding background counts and measurement time are represented, respectively [33]. The determined specific activity concentrations were finally used to evaluate diverse radiological hazard indices, imparting a complete assessment of potential exposure risks associated with the basaltic volcanic samples. Radium equivalent activity (Ra_eq_), internal Hazard Index (H_in_), external Hazard Index (H_ex_), absorbed dose rate (D_air_ in nGy h^−1^), annual effective dose (AED in mSv y^−1^), annual gonadal dose equivalent (AGDE, mSv y^−1^), and excess lifetime cancer risk (ELCR × 10^−3^) are derived metrics used to estimate levels of background radiation.

The radium equivalent activity (Ra_eq_) is the sum of the individual activities of the three naturally occurring radionuclides (^238^U, ^232^Th, and ^40^K) expressed as a single value, thus providing a means by which an affected person’s radiation exposure to these three isotopes may be assessed. Internationally, an acceptable maximum level of radium equivalent activity is 370 Bq kg^−1^. If the annual dose received by a person from this material is less than one millisievert (1 mSv), then it presents no significant risk to their health [34].

(3) $${Ra}_{eq}=A_{U}+1.43\;A_{Th}+0.077\;A_{K}$$

Calculating the external and internal hazard indices (H_ex_ and H_in_) for evaluating how exposure to radiation may harm human health is conducted using Equations (4) and (5), respectively [35,36]:

(4) $$H_{\mathrm{ex}}\;=\;\frac{A_{U}}{370}+\;\frac{A_{Th}}{259}+\frac{A_{K}}{4810}\leq 1$$

(5) $$H_{\mathrm{in}}=\frac{A_{U}}{185}+\frac{A_{Th}}{259}+\frac{A_{K}}{4810}\leq 1$$

H_ex_ and H_in_ should not exceed 1.0 [37,38].

The safe level of investigated materials containing radionuclides can be evaluated using the Iγ Index. The calculation of Iγ Values is derived from the relationship provided in Equation (6) [39]:

(6) $$I_{\gamma }\;=\;\frac{A_{U}}{150}+\;\frac{A_{Th}}{100}+\frac{A_{K}}{1500}\leq 1$$

To find the dose of gamma radiation that was received at a distance of one meter above ground from a source, the absorbed dose rate (also referred to as the D_air_) was calculated. The annual effective dose (AED) of both AED_out_ and AED_in_ can be estimated using occupancy factors of 0.2 for AED_out_ and 0.8 for AED_in_, and the methods used to calculate the annual effective dose are described in the subsequent sections [40,41].

D_air_ = 0.430 A_U_ + 0.666 A_Th_ + 0.042 A_K_ (7)

AED (mSv y^−1^) = D_air_ × (0.2 outdoor or 0.8 indoor) × 8760 h × 0.7 Sv/Gy × 10^−6^ (8)

The AGDE, which is an alternative form of measuring the hazard from radiation, is also involved in the yearly determination of the D_air_ from the whole body. For ^238^U, ^232^Th, and ^40^K, the AGDE is calculated based on the amount of gamma radiation emitted (Equation (9), [38]).

AGDE (mSv y^−1^) = 3.09*A*_U_ + 4.18*A*_Th_ + 0.314*A*_K_ (9)

The possibility of developing cancer due to exposure to gamma radiation over an individual’s lifetime (roughly 70 years) is known as the excess lifetime cancer risk (ELCR). The calculation for the ELCR is outlined in the (Formula (10), [42]), where the cancer risk factor is (RF = 0.05 Sv^−1^).

(10) $$ELCR\left(mSv/y\right)={AED}_{out}\times DL(70\;\mathrm{years})\times RF(0.05\;{\mathrm{Sv}}^{-1})$$

## 5. Results and Discussion

### 5.1. Radioactive Content Ratios

**Figure 4:**
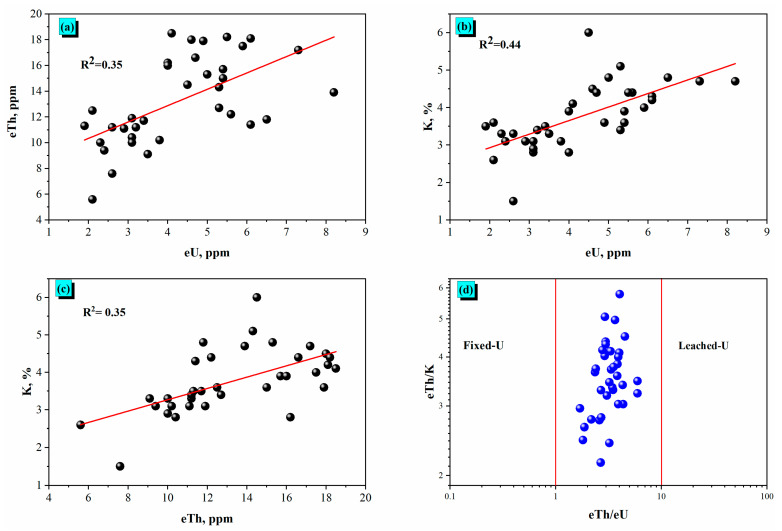
Correlations between the measured radionuclides. (**a**) eU vs. eTh (**b**) eU vs. K, % (**c**) eTh vs. K, % (**d**) eTh/eU vs. eTh/K.

Figure 4 reveals the correlation analysis among the radionuclide content eU (equivalent uranium), eTh (equivalent thorium), and K% (potassium) and shows key radioactive relationships indicative of their behavior inside basaltic volcanic samples. The data factors, derived from gamma-ray spectrometric measurements, display varying degrees of high-quality linear relationships for a few of the radionuclides.

#### 5.1.1. eU–eTh Relationship

Figure 4a presents a moderately high-positive correlation between eU and eTh (R^2^ = 0.35), suggesting co-enrichment underneath hydrothermal alteration and/or due to the presence of zircon. In basaltic rocks, this association normally displays uranium and thorium incorporation inside accessory minerals, inclusive of zircon. The moderate correlation can also reflect partial decoupling because of differing mobility under weathering or hydrothermal alteration conditions, with uranium being greater mobile.

#### 5.1.2. eU–K% Relationship

Figure 4b displays a slightly stronger correlation (R^2^ = 0.44) among eU and K% content. Sericite, as an alteration product of plagioclase, contains most of the potassium in basaltic volcanic rocks. This association factors into a shared geochemical conduct throughout redistribution via low-temperature alteration procedures. The link also indicates that uranium can be retained inside K-bearing levels or clay minerals formed through secondary alteration of volcanic rocks.

#### 5.1.3. eTh–K% Relationship

Figure 4c gives a comparable degree of correlation between eTh and K (R^2^ = 0.35). Like uranium, thorium can be found in zircon [25] and shows a weak correlation with potassium, related to hydrothermal alteration (sericitization). The consistency in R^2^ values between the eU–eTh and eTh–K pairs reinforces the secondary alteration.

#### 5.1.4. eTh/eU vs. eTh/K Ratio

Figure 4d describes a discrimination plot of the usage of eTh/eU versus eTh/K ratios to interpret uranium behavior. Most information factors cluster close to the middle, between the fixed-U and leached-U domain names, with eTh/eU ratios predominantly changing from 2 to 6. This distribution suggests slight uranium mobility, probably because of oxidative leaching, which is not unusual in subaerial volcanic terrains in which meteoric water interacts with U-bearing minerals. However, the absence of a strong shift toward both ceases, indicating restricted post-emplacement remobilization.

### 5.2. Radionuclide Concentrations and Their Hazards

The descriptive statistics for the specific activity concentrations of ^238^U, ^232^Th, and ^40^K in the basaltic volcanic rocks are summarized in Table 1. The findings reflect natural variation in radionuclide distribution influenced by the petrology of the host rocks. Uranium-238 (^238^U) concentrations range from 23 to 101 Bq kg^−1^, which has a mean ± SD of 53 ± 20 Bq kg^−1^. The moderate coefficient (CV = 37%) and weak positive skewness (+0.41) suggest a relatively wide dispersion with a trend against high values, indicating local enrichment due to magnetic discrimination or secondary remobilization under conditions of oxidation. Negative kurtosis (−0.41) reflects a flatter distribution from normal, the importance of a relatively balanced dataset without sharp peaks. Thorium-232 (^232^Th) suggests concentrations between 23 and 75 Bq kg^−1^, with an average of 54 ± 14 Bq kg^−1^. The CV of 25% and close to 0 skewness (−0.08) propose a symmetric distribution, indicating extra uniform thorium content within the basaltic samples. The slightly platykurtic nature (kurtosis = −0.78) also supports a wider, flatter spread in values, consistent with thorium’s geochemical immobility, frequently final locked in resistant accent minerals. Potassium-40 (^40^K) shows a concentration range from 470 to 1878 Bq kg^−1^, which has a mean of 1178 ± 269 Bq kg^−1^. The distribution of activity is approximately normal, indicated by skewness (+0.09), and shows less leptokurtosis (Kurtosis = +0.86), which sometimes suggests a trend towards central clustering with high values. 23% CV reflects moderate variability, which is usually attributed to the variation in K-bearing minerals that are feldspar and mica within volcanic rocks. The mean values of ^238^U, ^232^Th, and ^40^K in the basaltic samples are higher comparable to the worldwide average of 35, 45, and 412 Bq kg^−1^, respectively [3]. Moreover, these statistical styles highlight that uranium indicates the highest variability between the three different radionuclides, probably because of its sensitivity to alteration and redox conditions. In evaluation, thorium remains relatively strong, and potassium shows moderate variation, largely controlled through the mineralogical composition of the host rocks.

**Figure 5:**
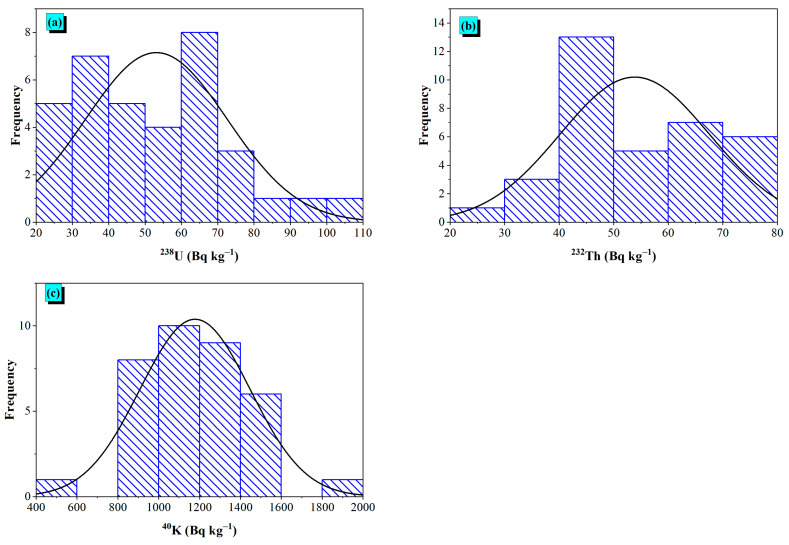
Frequency distribution of (**a**) ^238^U, (**b**) ^232^Th, and (**c**) ^40^K activity concentrations in the basaltic samples.

**Figure 6:**
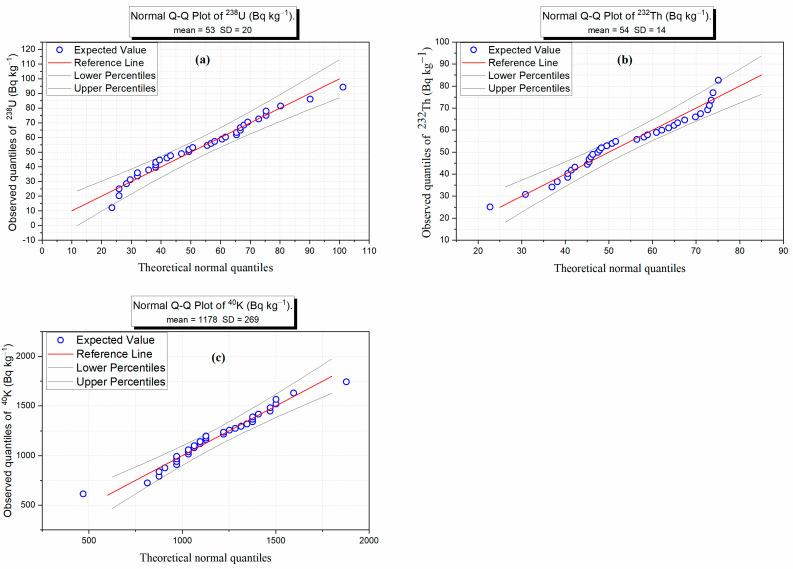
Q-Q plot of (**a**) ^238^U, (**b**) ^232^Th, and (**c**) ^40^K activity concentrations (Bq kg^−1^) in the basaltic samples.

The Gaussian function of frequency distributions in Figure 5 fits overlaying all histograms were generated using the sample means and standard deviations. These Gaussian function fits serve as a visual comparison between the empirical (observed) distribution of data and the theoretical Gaussian distribution. The frequency distributions (Figure 5) and Q-Q plots (Figure 6) display that ^238^U and ^232^Th observe near-normal distributions, with means of 53.1 Bq kg^−1^ and 53.8 Bq kg^−1^, respectively, suggesting hydrothermal alteration. On the other hand, ^40^K displays higher variability (mean = 1177.8 Bq kg^−1^), with deviations at extreme values, possibly because of its mobility in potassium-rich minerals. These patterns align with earlier correlations, reinforcing that thorium is a stable magmatic tracer, whilst uranium and potassium are more impacted via secondary procedures like hydrothermal activity or weathering.

While the weak skewness (0.41) and mild negative kurtosis (−0.41) demonstrate a slight deviation from a perfectly normal distribution, the results of the Shapiro–Wilk Test in Table 2 showed the data are still considered “statistically” normally distributed (*p* > 0.05). At a level of confidence of 95%, no statistically significant deviation from normal was identified for any of the three radionuclide data sets by the Shapiro–Wilk Test, with all three radionuclides having *p*-values greater than 0.05. Although the Shapiro–Wilk Test does not demonstrate normality, the combined evidence from numerical statistics and the visual assessment of frequency distributions and Q-Q plots all support the conclusion that these radionuclide data sets are sufficiently representative of normal distribution, and therefore meet the normal distribution assumption of parametric analyses.

The activity concentrations of ^238^U, ^232^Th, and ^40^K in the basaltic samples are compared to previous investigations (Table 3) to determine their mean values. This comparison shows that the geological characteristics of the analyzed sites affect the activity concentrations of these elements.

### 5.3. Radiological Hazard Assessment

Table S1 presents the radiological health risks related to exposure to ^238^U, ^232^Th, and ^40^K radionuclides in the basaltic samples. Several radiological hazard indices have been calculated.

#### 5.3.1. Radium Equivalent Activity (Ra_eq_)

The Ra_eq_ values in the basaltic samples vary from 112 to 303 Bq kg^−1^, with a mean of 221 Bq kg^−1^. This parameter gives a single index to evaluate the specific activities of ^238^U, ^232^Th, and ^40^K. All samples fall below the recommended safety limit of 370 Bq kg^−1^ set by [52,53], suggesting that the gamma radiation threat from these basaltic materials is within permissible levels for constructing materials.

#### 5.3.2. Hazard Indices (H_ex_ and H_in_)

The external hazard index (H_ex_) changes from 0.30 to 0.82, and the internal hazard index (H_in_) from 0.39 to 1.07, with a mean value of 0.60 and 0.74, respectively. For radiation safety, both indices should be less than unity. While maximum samples follow this criterion, a few samples (S13, S14, S15, S33) approach or slightly exceed 1.0 for H_in_, indicating a potential radiological risk commonly from radon and its progeny in enclosed environments.

#### 5.3.3. Gamma Index (Iγ)

The gamma index, Iγ, used to evaluate suitability for constructing materials, varies from 0.42 to 1.14. The mean value of 0.84 is under the threshold value of 1.0, suggesting that almost all of the basaltic are marginally perfect to be used in production. However, samples with Iγ > 1 (S13–S15, S28, S30, S33) can also require exclusion or specific management before utility basaltic in residential buildings.

#### 5.3.4. Absorbed Dose Rate and Effective Doses

The absorbed dose charge in air (D_air_) levels range from 53 to 144 nGy h^−1^, with a mean of 105 nGy h^−1^, which is higher than the global average of 59 nGy h^−1^ [38]. This shows a pretty expanded external exposure to terrestrial gamma radiation. The annual effective dose (AED) values are also expanded, where AED_out_ ranges from 0.06 to 0.18 mSv y^−1^ (mean: 0.13 mSv y^−1^); moreover, AED_in_ varies from 0.26 to 0.71 mSv y^−1^ with a mean of 0.52 mSv y^−1^. Both values are in the acceptable limit of 0.07 and 0.41 mSv y^−1^ for outdoor and indoor, respectively [38], even though some indoor doses approach the upper bound, emphasizing the need for ventilation if such basaltic materials are used indoors.

#### 5.3.5. Annual Gonadal Dose Equivalent (AGDE) and ELCR

The AGDE ranges from 0.38 to 1.03 mSv y^−1^, with a mean of 0.76 mSv y^−1^. This parameter displays the genetic influences of radiation and, in addition, confirms a relatively high exposure from basaltic material samples. The excess lifetime cancer risk (ELCR) varies between 0.23 × 10^−3^ and 0.62 × 10^−3^, with an average of 0.45 × 10^−3^. While those values remain below the worldwide average (2.9 × 10^−3^) [42], they reflect a moderate level of long-term radiological risk, especially when exposure is prolonged or occurs in poorly ventilated indoor environments.

The Pearson correlation evaluation in Table 4 is performed to assess the linear relationships among the activity concentrations of the primordial radionuclides (^238^U, ^232^Th, and ^40^K) and the derived radiological danger indices (Ra_eq_, H_in_, H_ex_, Iγ, D_air_, AED_out_, AED_in_, AGDE, and ELCR). The effects are supplied in the correlation matrix and suggest several statistically significant and strong positive relationships between the parameters. The correlation coefficient between ^238^U and ^232^Th was slight (r = 0.60), suggesting hydrothermal alteration. A higher correlation turned into determined between ^238^U and ^40^K (r = 0.67), indicating a stronger association between uranium and potassium-bearing phases, consisting of feldspar or mica, which may be residual from intermediate volcanic differentiation. ^232^Th additionally confirmed a slight correlation with ^40^K (r = 0.59), further assisting the inference that these elements are co-enriched in positive mineralogical components of the volcanic rocks. All three radionuclides (^238^U, ^232^Th, and ^40^K) displayed strong positive correlations with the radiological hazard parameters. Notably, ^238^U had a very strong correlation with the internal danger index H_in_ (r = 0.93) and with Ra_eq_, H_ex_, and ELCR (r = 0.87 each), indicating that uranium is a major contributor to the general radiological risk inside the studied samples. This can be attributed to uranium’s higher specific activity and potential mobility in volcanic settings, inspired by the aid of hydrothermal fluids. Similarly, ^40^K established the strongest average correlations with radiological parameters, especially with AGDE (r = 0.91), and with Iγ, D_air_, and both AED_out_ and AED_in_ (r = 0.90 each). These studies recommend that potassium contributes considerably to the external gamma dose and long-term radiological impact, consistent with its abundance in intermediate volcanic rocks. ^232^Th also showed strong correlations with all risk indices, mainly Ra_eq_ and AGDE (r = 0.84 and r = 0.82, respectively), asserting its role in radiological exposure despite its decreased mobility in comparison to uranium. Overall, the strong correlations among ^238^U, ^232^Th, and ^40^K and the derived risk indices mirror their cumulative and overlapping contributions to environmental radioactivity.

The hierarchical cluster evaluation (HCA) illustrated in Figure S1 presents a comprehensive view of the relationships amongst certainly occurring radionuclides (^238^U, ^232^Th, and ^40^K) and related radiological threat indices in basaltic volcanic samples. The dendrogram identifies three primary clusters that mirror the underlying radiological impact of those radionuclides in basaltic geological settings. The first and most dominant cluster consists of ^238^U and ^40^K in conjunction with a suite of radiological indices consisting of radium equal interest (Ra_eq_), external threat index (H_ex_), internal risk index (H_in_), absorbed dose charge in air (D_air_), annual effective dose (AED_in_ and AED_out_), annual gonadal dose equivalent (AGDE), excess lifetime cancer chance (ELCR), and the gamma index (Iγ). This strong association shows that ^238^U and ^40^K are the essential contributors to radiation risks in the basaltic rocks studied. Their clustering with multiple hazard indices indicates that their activity concentrations significantly affect the overall radiological danger in those volcanic materials. This is regular with the mineral composition of basalt, which commonly includes potassium-bearing feldspars and trace amounts of uranium-bearing minerals, thereby raising ^40^K and ^238^U phases. In comparison, ^232^Th establishes a separate cluster, displaying a weaker statistical association with the hazard indices. This separation can be attributed to thorium concentration decrease in basaltic rocks in comparison to their more felsic counterparts. Its distinct clustering shows that ^232^Th contributes much less to the range in hazard indices inside these samples, even though it nevertheless plays a role in long-time-period radiological exposure due to its long half-life and radiotoxicity. Additionally, a sub-cluster comprising Ra_eq_, H_ex_, D_air_, AGDE, and Iγ reflects the near mathematical and conceptual relationships amongst these indices, as they are all directly impacted through radionuclide activity concentrations.

The primary factor evaluation (PCA) presented in Figure S2 affords valuable insights into the relationships among radionuclides ^238^U, ^232^Th, and ^40^K and radiological hazard indices in basaltic volcanic samples. The PCA biplot suggests that the first component (Factor 1) accounts for a dominant 92.84% of the total variance, while the second component (Factor 2) contributes only 3.94%, indicating that most of the variability within the dataset may be efficiently interpreted alongside the first axis. A positive strong correlation is determined among ^238^U and ^40^K, and the majority of the radiological hazard indices, which include Ra_eq_, H_in_, H_ex_, Iγ, D_air_, AED_out_, AED_in_, AGDE, and ELCR. These variables cluster tightly on the negative side of Factor 1, indicating that ^238^U and ^40^K are the essential contributors to the radiological impact of the studied basaltic samples. This displays the geochemical nature of basalts, which typically contain potassium-bearing feldspars and uranium-rich accessory minerals, making ^40^K and ^238^U large assets of natural radioactivity. Their strong association with risk indices highlights their key position in controlling dose-related parameters in volcanic environments. In the assessment, ^232^Th appears inaccessible and positioned similarly away from the cluster of indices, with a lower loading on Factor 1 and a moderate negative impact on Factor 2. This separation implies a weaker correlation between ^232^Th and the hazard indices in the basaltic context. Thorium is generally much less mobile and tends to be much less uniformly distributed in basaltic rocks, which may also explain its decreased statistical effect at the derived dose indices. Generally, the PCA evaluation corroborates the results from the cluster analysis and confirms that ^238^U and ^40^K dominate the radiological profile of the basaltic volcanic samples. These findings reinforce the significance of these radionuclides in hazard evaluation and radiological monitoring, even as also suggesting that ^232^Th, although present, contributes more variably and should be treated with distinct consideration in environmental assessments.

## 6. Conclusions

This study provides an integrated assessment of the petrographic characteristics, natural radioactivity contents, and associated radiological hazards in basaltic volcanic rocks. Petrographic examination identified obvious mineralogical variations, with the basaltic samples dominated by plagioclase, olivine, and pyroxene. The presence of secondary alteration minerals such as chlorite, epidote, and sericite indicates rigorous secondary processes acting in these volcanic rocks. Radiometric measurements indicated high levels of natural radionuclides, with average activity concentrations of 53 ± 20 Bq kg^−1^ for ^238^U, 54 ± 14 Bq kg^−1^ for ^232^Th, and 1178 ± 269 Bq kg^−1^ for ^40^K, all higher than worldwide means. Statistical analysis indicated normal distributions for the radionuclides, with uranium showing the highest variability. Correlation analysis indicated moderate correlations between eU-eTh and eTh-K%, and a high correlation between eU-K%, indicating uranium’s affinity for potassium-bearing minerals. The mean radium equivalent activity (Ra_eq_) was 221 Bq kg^−1^, less than the suggested 370 Bq kg^−1^. Both the external and internal hazard indices (H_ex_ and H_in_) were mostly less than unity, but there were samples above the H_in_ threshold, suggesting potential indoor radon hazards. The absorbed dose rate in air at 1 m above the ground (105 nGy h^−1^), annual effective dose (0.52 mSv y^−1^ indoors), and excess lifetime cancer risk (0.45 × 10^−3^) were higher than the world averages but within safe limits for long-term exposure. Multivariate analyses, including hierarchical cluster analysis and principal component analysis, consistently picked out ^238^U and ^40^K as the primary contributors to radiological hazards, while ^232^Th had lower correlations due to its geochemical stability. However, the results highlight the need for caution when these materials are used in the construction industry, particularly in residential areas.

## Figures and Tables

**Table 1:** Descriptive statistical analysis of ^238^U, ^232^Th, and ^40^K concentrations in 35 samples of basaltic volcanic rocks.

	N	Mean	SD	Min	Max	Skewness	Kurtosis	CV, %
^238^U (Bq kg^−1^)	35	53	20	23	101	0.41	−0.41	37
^232^Th (Bq kg^−1^)	35	54	14	23	75	−0.08	−0.78	25
^40^K (Bq kg^−1^)	35	1178	269	470	1878	0.09	0.86	23

SD = Standard deviation.

**Table 2:** Shapiro–Wilk test results for normality of radionuclide ^238^U, ^232^Th, and ^40^K concentrations.

	N	Statistic	*p*-Value
^238^U	35	0.96	0.29
^232^Th	35	0.95	0.15
^40^K	35	0.98	0.63

**Table 3:** Comparison of ^238^U, ^232^Th, and ^40^K activity concentration in the basaltic samples at Monqul area with numerous world studies.

Country	^238^U	^232^Th	^40^K	References
	Bq kg^−1^	Bq kg^−1^	Bq kg^−1^	
Egypt	53	54	1178	Present study
Egypt	46.39	65.76	1186.45	[43]
Egypt	370	68	1355	[44]
Bosnia and Herzegovina	119	96	1070	[45]
Ethiopia	51.9	68.32	220	[46]
Saudi Arabia	28.58	37.04	960.48	[47]
India	43.2	71.2	895.2	[48]
Turkia	77.5	6.3	140	[49]
Vietnam	172	562	849	[50]
Nigeria	18.15	42.86	570.91	[51]
Worldwide average	35	45	412	[3]

**Table 4:** Pearson correlation matrix between radionuclides and radiological parameters.

	^238^U	^232^Th	^40^K	Ra_eq_	H_in_	H_ex_	Iγ	D_air_	AED_out_	AEDin	AGDE	ELCR
^238^U	1.00	0.60	0.67	0.87	0.93	0.87	0.86	0.87	0.87	0.87	0.86	0.87
^232^Th	//	1.00	0.59	0.84	0.80	0.84	0.83	0.82	0.82	0.82	0.82	0.82
^40^K	//	//	1.00	0.88	0.84	0.88	0.90	0.90	0.90	0.90	0.91	0.90

## Data Availability

The datasets used and/or analyzed during the current study are available from the corresponding author on reasonable request.

## Supplementary Materials

The following supporting information can be downloaded at: https://www.mdpi.com/article/10.3390/toxics14010015/s1, Table S1. Radiological hazard indices for basaltic volcanic samples. Figure S1. Clustering analysis of 238U, 232Th, and 40K activity concentrations with radiological hazard indices in the basaltic volcanic samples. Figure S2. Principal component analysis (PCA) of 238U, 232Th, and 40K activity concentrations with radiological hazard indices in the basaltic samples.

## References

[1] Söğüt Ö., Zorer Ö.S., Reyhanlıoğlu H. (2018). Radiological Hazard Assessment of Natural Gross Radioactivity in Some Cosmetic Products. Environ. Forensics.

[2] Salbu B., Lind O.C. (2020). Analytical Techniques for Charactering Radioactive Particles Deposited in the Environment. J. Environ. Radioact..

[3] UNSCEAR (2010). Sources and Effects of Ionizing Radiation—Exposures of The Public and Workers From Various Sources of Radiation—UNSCEAR 2008 Report.

[4] Abdel Wahed A.A.A., Ali K.G., Khalil M.M.A., Abdel Gawad A.E. (2012). Dokhan Volcanics of Gabal Monqul Area, North Eastern Desert, Egypt: Geochemistry and Petrogenesis. Arab. J. Geosci..

[5] Uyanik N.A., Öncü Z., Uyanik O., Bozcu M., Akkurt İ., Günoğlu K. (2015). Distribution of Natural Radioactivity from 40 K Radioelement in Volcanics of Sandıklı-Şuhut (Afyon) Area. Acta Phys. Pol. A.

[6] Shahin H.A.A., Masoud M.S., Bayoumi M.M. (2016). Geology, Geochemistry and Radioactivity of Granitic and Volcanic Rocks at Hadarba Area, South Eastern Desert, Egypt. Int. Res. J. Geol. Min..

[7] (2019). Atlas of Magmatic Rocks. Atlas of Rocks. https://www.atlas-hornin.sk/en/home.

[8] Harangi S. (2001). Neogene to Quaternary Volcanism of the Carpathian-Pannonian Region-A Review. Acta Geol. Hung..

[9] Bonewitz R. (2012). Rocks and Minerals.

[10] Rashwan M.A., Lasheen E.S.R., Azer M.K. (2023). Thermal and Physico-Mechanical Evaluation of Some Magmatic Rocks at Homrit Waggat Area, Eastern Desert, Egypt: Petrography and Geochemistry. Bull. Eng. Geol. Environ..

[11] Abdel-Rahman A.M., Gahlan H.A., Abdel Latif M.L., Elyaseer M.H., Price J.B., Azer M.K. (2025). Mineral Chemistry, ASTER Hydrothermal Alteration Mapping, and Geotechnical Characterization of Granitic Rocks of the Arabian-Nubian Shield; A Case Study from Wadi El-Hima, South Eastern Desert, Egypt. J. Afr. Earth Sci..

[12] Eliwa H.A., El-Bialy M.Z., Murata M. (2014). Edicaran Post-Collisional Volcanism in the Arabian-Nubian Shield: The High-K Calc-Alkaline Dokhan Volcanics of Gabal Samr El-Qaa (592±5Ma), North Eastern Desert, Egypt. Precambrian Res..

[13] Moghazi A.M. (2003). Geochemistry and Petrogenesis of a High-K Calc-Alkaline Dokhan Volcanic Suite, South Safaga Area, Egypt: The Role of Late Neoproterozoic Crustal Extension. Precambrian Res..

[14] Novoselov A.A., Silva D., de Souza Filho C.R. (2020). Authigenic Titanite in Weathered Basalts: Implications for Paleoatmospheric Reconstructions. Geosci. Front..

[15] Ramos F.C., Wolff J.A., Tollstrup D.L. (2004). Measuring 87Sr/86Sr Variations in Minerals and Groundmass from Basalts Using LA-MC-ICPMS. Chem. Geol..

[16] Sun S.-s., McDonough W.F. (1989). Chemical and Isotopic Systematics of Oceanic Basalts: Implications for Mantle Composition and Processes. Geol. Soc. Lond. Spec. Publ..

[17] Chen X., Zhang Y., Hui D., Chen M., Wu Z. (2017). Study of Melting Properties of Basalt Based on Their Mineral Components. Compos. B Eng..

[18] Irvine T.N., Baragar W.R.A. (1971). A Guide to the Chemical Classification of the Common Volcanic Rocks. Can. J. Earth Sci..

[19] Ragab A.A. (2010). Geochemical Behavior of Rare Earth Elements in Hydrothermally Altered Rhyolite of Um Safi Volcanics, Central Eastern Desert, Egypt. Al-Azhar Bull. Sci..

[20] Maithani P.B., Srinivasan S. (2011). Felsic Volcanic Rocks, a Potential Source of Uranium—An Indian Overview. Energy Procedia.

[21] Cunningham C.G., Rasmussen J.D., Steven T.A., Rye R.O., Rowley P.D., Romberger S.B., Selverstone J. (1998). Hydrothermal Uranium Deposits Containing Molybdenum and Fluorite in the Marysvale Volcanic Field, West-Central Utah. Miner. Depos..

[22] Hanfi M.Y., Abdel Gawad A.E., Ali K.G., Abu-Donia A., Alsafi K.G., Khafaji M.A., Albahiti S.K., Alqahtani M.S., Khalil M., Abdel Wahed A.A. (2022). Environmental Risk Assessment Associated with Acidic Volcanics in Egypt. Appl. Radiat. Isot..

[23] Eliwa H.A., Kimura J.-I., Itaya T. (2006). Late Neoproterozoic Dokhan Volcanics, North Eastern Desert, Egypt: Geochemistry and Petrogenesis. Precambrian Res..

[24] Sherif H.M. Petrography, Geochemistry and K–Ar Ages of Paleogene Basalts, West Shalatein, South Eastern Desert, Egypt. Proceedings of the the Fifth International Conference on the Geology of Africa.

[25] Shahin H.A.A., Masoud M.S. (2013). Geology and Geochemistry of Tertiary Basalt in South Wadi Hodein Area, South Eastern Desert, Egypt. Arab. J. Geosci..

[26] El-Bialy M.Z. (2010). On the Pan-African Transition of the Arabian-Nubian Shield from Compression to Extension: The Post-Collision Dokhan Volcanic Suite of Kid-Malhak Region, Sinai, Egypt. Gondwana Res..

[27] Obeid M.A. (2006). The Pan-African Arc-Related Volcanism of the Wadi Hodein Area, South Eastern Desert, Egypt: Petrological and Geochemical Constraints. J. Afr. Earth Sci..

[28] Liégeois J.-P., Stern R.J. (2010). Sr–Nd Isotopes and Geochemistry of Granite-Gneiss Complexes from the Meatiq and Hafafit Domes, Eastern Desert, Egypt: No Evidence for Pre-Neoproterozoic Crust. J. Afr. Earth Sci..

[29] Stern R.J., Hedge C.E. (1985). Geochronologic and Isotopic Constraints on Late Precambrian Crustal Evolution in the Eastern Desert of Egypt. Am. J. Sci..

[30] Hegab M.A.E.-R., Salem S.M. (2021). Mineral-Bearing Alteration Zones at Gebel Monqul Area, North Eastern Desert, Egypt, Using Remote Sensing and Gamma-Ray Spectrometry Data. Arab. J. Geosci..

[31] Abdel Gawad A.E., Ali K.G., Wahed A.A.A., Alsafi K., Khafaji M., Albahiti S., Khalil M., Masoud M.S., Hanfi M.Y. (2022). Excess Lifetime Cancer Risk Associated with Granite Bearing Radioactive Minerals and Valuable Metals, Monqul Area, North Eastern Desert, Egypt. Materials.

[32] Botros N.S., Wetait M.A. (1997). Possible Porphyry Copper Mineralization in South Um Monqul, Eastern Desert, Egypt. Egypt J. Geol..

[33] Abedin M.J., Karim M.R., Hossain S., Deb N., Kamal M., Miah M.H.A., Khandaker M.U. (2019). Spatial Distribution of Radionuclides in Agricultural Soil in the Vicinity of a Coal-Fired Brick Kiln. Arab. J. Geosci..

[34] Shuaibu H.K., Khandaker M.U., Alrefae T., Bradley D.A. (2017). Assessment of Natural Radioactivity and Gamma-Ray Dose in Monazite Rich Black Sand Beach of Penang Island, Malaysia. Mar. Pollut. Bull..

[35] Sivakumar S., Chandrasekaran A., Senthilkumar G., Suresh Gandhi M., Ravisankar R. (2018). Determination of Radioactivity Levels and Associated Hazards of Coastal Sediment from South East Coast of Tamil Nadu with Statistical Approach. Iran. J. Sci. Technol. Trans. A Sci..

[36] Yasmin S., Barua B.S., Khandaker M.U., Kamal M., Rashid A., Sani S.F.A., Ahmed H., Nikouravan B., Bradley D.A. (2018). The Presence of Radioactive Materials in Soil, Sand and Sediment Samples of Potenga Sea Beach Area, Chittagong, Bangladesh: Geological Characteristics and Environmental Implication. Results Phys..

[37] Yalcin S., Gurler O. (2007). The Radioactivity Measurements in Soil, Coal and Water in South Marmara Region of Turkey. Radiat. Meas..

[38] UNSCEAR (2000). Exposures from Natural Radiation Sources (Annex B). Sources and Effects of Ionizing Radiation.

[39] Devanesan E., Chandramohan J., Senthilkumar G., Harikrishnan N., Suresh Gandhi M., Kolekar S.S., Ravisankar R. (2020). Natural Radioactivity Concentrations and Dose Assessment in Coastal Sediments along the East Coast of Tamilnadu, India with Statistical Approach. Shengtai Xuebao/Acta Ecol. Sin..

[40] Baykara O., Karatepe Ş., Doǧru M. (2011). Assessments of Natural Radioactivity and Radiological Hazards in Construction Materials Used in Elazig, Turkey. Radiat. Meas..

[41] Abdel Gawad A.E., Masoud M.S., Khandaker M.U., Hanfi M.Y. (2024). Radiological Hazards Assessment Associated with Granitoid Rocks in Egypt. Nucl. Eng. Technol..

[42] Qureshi A.A., Tariq S., Kamal U., Manzoor S., Calligaris C., Waheed A. (2014). Evaluation of Excessive Lifetime Cancer Risk Due to Natural Radioactivity in the Rivers Sediments of Northern Pakistan. J. Radiat. Res. Appl. Sci..

[43] Darwish D.A.E., Abul-Nasr K.T.M., El-Khayatt A.M. (2015). The Assessment of Natural Radioactivity and Its Associated Radiological Hazards and Dose Parameters in Granite Samples from South Sinai, Egypt. J. Radiat. Res. Appl. Sci..

[44] Abdel Gawad A.E., Hanfi M.Y., Alqahtani M.S., Ramadan A.A. (2025). Geochemical Features and Radiological Risk Assessment of Wadi El-Regeita Granites, South Central Sinai, Egypt. Radiat. Phys. Chem..

[45] Kuzmanović P., Petrović L.F., Hansman J., Mrđa D., Forkapić S., Velimirović D., Demirhan K., Radić J.K. (2024). Natural Radioactivity and Technological Properties of Kaolinized Granite from the Motajica Mine, Bosnia and Herzegovina. Constr. Build. Mater..

[46] Regassa T.N., Raba G.A., Chekol B.M., Kpeglo D.O. (2023). Assessment of Natural Radioactivity and Associated Radiological Risks from Soils of Hakim Gara Quarry Sites in Ethiopia. Heliyon.

[47] Salama M.A., Korany K.A., Ezzeldien M. (2025). Marble and Granite as Natural Radiation Sources: Risk Assessment and Their Impact on Health and the Environment. J. Radiat. Res. Appl. Sci..

[48] Poojitha C.G., Sahoo B.K., Ganesh K.E., Prajith R., Kumbhar D.H., Sapra B.K. (2024). Assessment of Primordial Radionuclides and Radon Exhalation in Granitic Terrain of Bengaluru, India. Nucl. Instrum. Methods Phys. Res. B.

[49] Turhan S., Aykamis A.S., Kilic A.M. (2009). Natural Radionuclide Content and Radiological Hazard Associated with Usage of Quartzite Sand Samples from Ovacik-Silifke-Mersin Open Pit as Building Material in Turkey. Radiat. Prot. Dosim..

[50] Duong N.T., Van Hao D., Bui V.L., Duong D.T., Phan T.T., Le Xuan H. (2021). Natural Radionuclides and Assessment of Radiological Hazards in MuongHum, Lao Cai, Vietnam. Chemosphere.

[51] Orosun M.M., Usikalu M.R., Oyewumi K.J., Adagunodo T.A. (2019). Natural Radionuclides and Radiological Risk Assessment of Granite Mining Field in Asa, North-Central Nigeria. MethodsX.

[52] NEA-OECD (1979). Exposure to Radiation from Natural Radioactivity in Building Materials. Report by NEA Group of Experts of the Nuclear Energy Agency.

[53] Beretka J., Mathew P.J. (1985). Natural Radioactivity of Australian Building Materials, Industrial Wastes and By-Products. Health Phys..

