# Supplementary material for: Environmental Investigation of Natural Radioactivity and Health Risk Assessment in Basaltic Volcanic Building Materials
Source: Toxics. 2025 Dec 22;14(1):15. doi: 10.3390/toxics14010015 (PMC12845954; doi:10.3390/toxics14010015)
Supplement: Supplementary file 1 [file toxics-14-00015-s001.zip › toxics-4017300-supplementary.pdf]

Supplementary material (SI)

Table **S1**. Radiological hazard indices for basaltic volcanic samples.

Samples	Ra_{eq} Bq kg^{-1}	H_{in}	H_{ex}	I_γ	D_{air} (nGy/h)	AED_{out} (mSv y^{-1})	AED_{in} (mSv y^{-1})	AGDE (mSv y^{-1})	ELCR
S1	112	0.39	0.30	0.42	53	0.06	0.26	0.38	0.23
S2	121	0.40	0.33	0.47	59	0.07	0.29	0.43	0.25
S3	166	0.55	0.45	0.63	79	0.10	0.39	0.57	0.34
S4	176	0.59	0.47	0.67	85	0.10	0.42	0.61	0.36
S5	177	0.56	0.48	0.68	85	0.10	0.42	0.61	0.36
S6	166	0.53	0.45	0.64	80	0.10	0.39	0.58	0.34
S7	186	0.61	0.50	0.71	89	0.11	0.44	0.65	0.38
S8	185	0.57	0.50	0.72	89	0.11	0.44	0.65	0.38
S9	194	0.64	0.52	0.74	93	0.11	0.46	0.67	0.40
S10	211	0.70	0.57	0.79	98	0.12	0.48	0.70	0.42
S11	252	0.86	0.68	0.95	119	0.15	0.59	0.86	0.51
S12	245	0.87	0.66	0.93	118	0.14	0.58	0.85	0.51
S13	271	0.93	0.73	1.02	128	0.16	0.63	0.92	0.55
S14	282	0.96	0.76	1.06	133	0.16	0.65	0.95	0.57
S15	303	1.06	0.82	1.14	144	0.18	0.71	1.03	0.62
S16	166	0.55	0.45	0.63	79	0.10	0.39	0.57	0.34
S17	159	0.51	0.43	0.61	77	0.09	0.38	0.56	0.33
S18	175	0.57	0.47	0.67	84	0.10	0.41	0.60	0.36
S19	181	0.62	0.49	0.69	86	0.11	0.42	0.62	0.37
S20	182	0.60	0.49	0.69	87	0.11	0.43	0.62	0.37
S21	221	0.77	0.60	0.83	105	0.13	0.52	0.75	0.45
S22	241	0.83	0.65	0.90	114	0.14	0.56	0.81	0.49
S23	251	0.84	0.68	0.94	118	0.14	0.58	0.84	0.51
S24	236	0.77	0.64	0.90	112	0.14	0.55	0.81	0.48

S25	246	0.85	0.66	0.94	118	0.15	0.58	0.85	0.51
S26	264	0.93	0.71	1.01	128	0.16	0.63	0.92	0.55
S27	271	0.91	0.73	1.04	131	0.16	0.64	0.95	0.56
S28	270	0.88	0.73	1.02	128	0.16	0.63	0.92	0.55
S29	260	0.86	0.70	0.99	124	0.15	0.61	0.89	0.53
S30	284	0.92	0.77	1.11	138	0.17	0.68	1.01	0.59
S31	266	0.89	0.72	1.02	128	0.16	0.63	0.92	0.55
S32	257	0.83	0.69	0.97	121	0.15	0.60	0.87	0.52
S33	295	1.07	0.80	1.11	141	0.17	0.69	1.01	0.61
S34	280	0.94	0.76	1.05	132	0.16	0.65	0.95	0.57
S35	173	0.53	0.47	0.67	83	0.10	0.41	0.61	0.36
Mean	221	0.74	0.60	0.84	105	0.13	0.52	0.76	0.45
SD	52	0.19	0.14	0.19	25	0.03	0.12	0.18	0.11
Min	112	0.39	0.30	0.42	53	0.06	0.26	0.38	0.23
Max	303	1.07	0.82	1.14	144	0.18	0.71	1.03	0.62

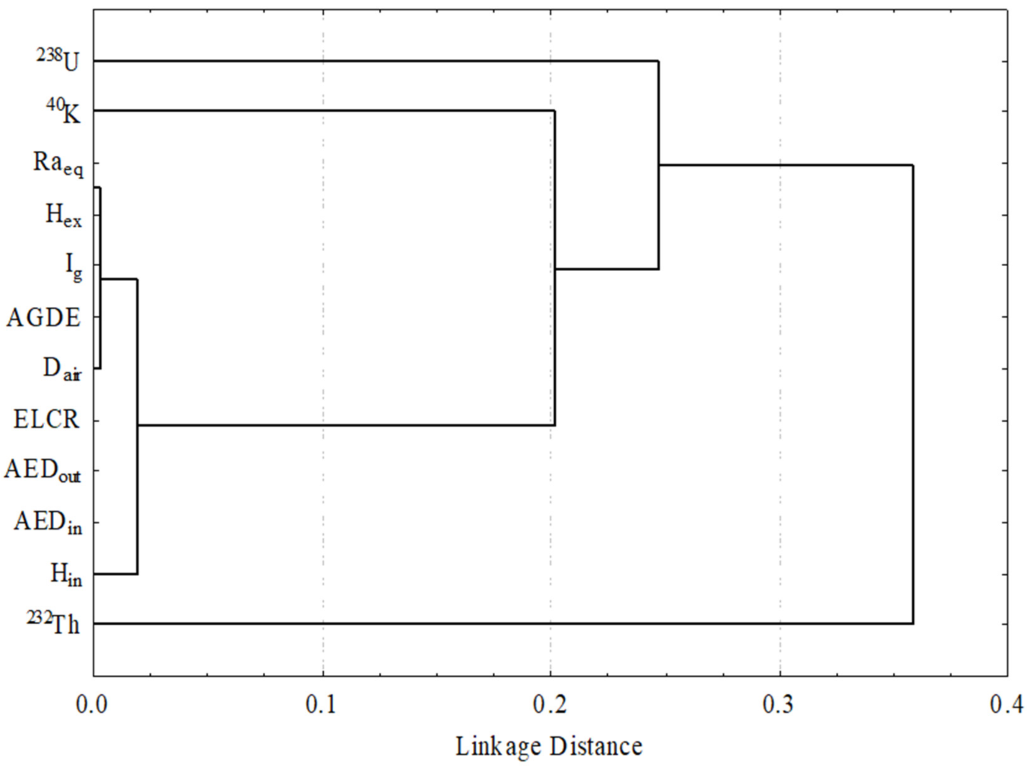

Figure S1. Clustering analysis of ^{238}U, ^{232}Th, and ^{40}K activity concentrations with radiological hazard indices in the basaltic volcanic samples.

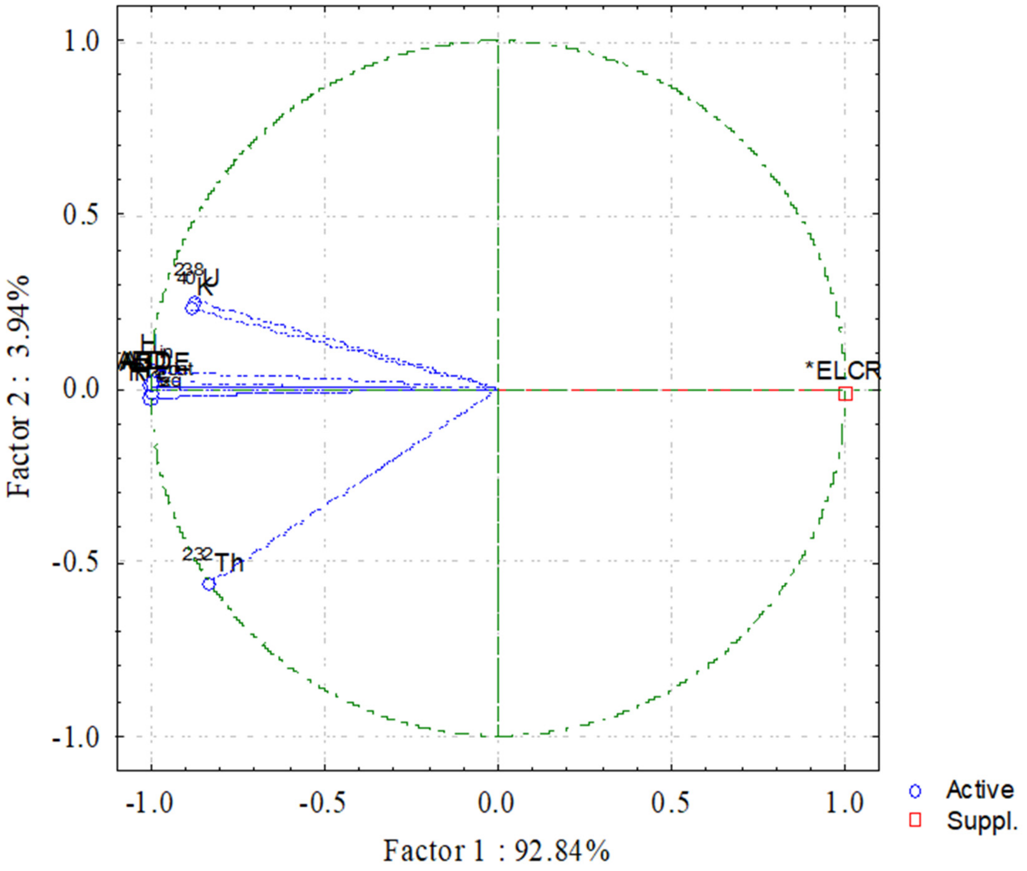

Figure S2. Principal component analysis (PCA) of ^{238}U, ^{232}Th, and ^{40}K activity concentrations with radiological hazard indices in the basaltic samples.
